# Legal Notes for Institutional Workers

**Published:** 1912-04-06

**Authors:** 


### LEGAL NOTES FOR INSTITUTIONAL WORKERS.

(The Editor will be pleased to answer Legal queries in this column.)

### Exposure of Infectious Persons.

Having regard to the vigorous attempts recently made to inculcate a knowledge of the simple laws of health, educated persons might be expected to protect the public as far as possible from exposure to infection. Yet the records of the courts occasionally show that offences are committed by persons who cannot plead ignorance in extenuation. Instance a recent case at Withnell. It appeared that the landlord of an inn, who was suffering from scarlet fever, was instructed to isolate himself. Notwithstanding the warning, he went to the shop of a tobacconist at Chorley. Noticing the state of his hands, the shopman inquired whether he had eczema. "Oh no," was the reply, "I've got scarlet fever, but say nowt about it or I shall get six months. But thou ought to see my body; it's coming off in lumps." Small wonder that there are 15 cases of scarlet fever in the district. The magistrates took a serious view of the offence, and fined the defendant £3 and costs. The most they could have fined him was £5, and none will say that the punishment was excessive. It is a strange anomaly in our law that a man who deliberately endangers the health of a district can only be fined, while the starving tramp who snatches a loaf can be sent to prison.

### The Position of a Locum Tenens.

Although it has been the fashion to employ the *locum tenens* for a great number of years, many doubtful points seem to arise with regard to the legal relations between him and his principal. In a case recently heard at the Clerkenwell County Court a *locum* sued for breach of contract in the following circumstances. He was engaged to act from November 30 to January 16, at a salary of £4 4s. per week with board and lodging. On December 15 he gave up work through illness; but when he recovered, his principal refused to employ him further. The action was accordingly brought to recover the salary which he would have earned during the rest of the period, and the value of the board and lodging. A transfer agent gave evidence to the effect that, according to the custom of the profession, a notice of 48 hours would terminate such an engagement. The defendant, who said he had been engaging *locum tenentes*, corroborated this view. The plaintiff, whose contention on the question of notice does not appear to be clearly stated in the *Times* report, argued that he was in the position of a servant, and that being so, it was unfair that he should have to bear the whole expense of illness. Judge Roberts, in giving judgment, pointed out that the defendant had had to employ another *locum*, and that being so, it was difficult to see how he could be made to pay the plaintiff as well. The claim was therefore dismissed. The moral of the case is that when the young practitioner is seeking employment of this kind, the contingency of illness should be specially provided for in a written agreement.

### Master's Liability for a Servant's Illness.

The case which we have commented on above leads naturally to the consideration of a further question—namely, Is the master liable for a servant's illness? Although the Chancellor of the Exchequer is responsible for the statement that the answer to this conundrum is doubtful, his doubt was political, not legal. In law the master is not liable to pay his servant wages when ill, nor is he liable to pay for medicine or medical attendance. In practice, of course, he frequently assumes responsibility for the bill of the doctor who has attended his cook, while his wife continues to pay the weekly wages; but legally there is no responsibility. Although the point is never likely to arise having regard to the care which is taken of members of the staff of a hospital, yet it is plain that hospital authorities are under no compulsion to provide medicines and attendance for any member of the staff who happens to be laid low by illness.

### The Manchester Fever Hospital Case.

A judgment which profoundly affects all managers of hospitals for infectious diseases was delivered in the Court of Appeal on March 30, reversing the previous decision of the Judge of the Lancaster County Court. The applicant in the suit was employed as a porter in the Scarlet Fever Hospital at Manchester. He contracted influenza, and returned to residence in the hospital after several weeks of absence. On a certain date, about a week after resuming work, he cleaned the mortuary, and about three or four hours afterwards was sick; he continued to suffer from sickness, sore throat, and headache for four more days, when a definite diagnosis of scarlet fever was made. Nephritis supervened, and compensation was claimed under the Workmen's Compensation Act. The County Court Judge held that the scarlet fever was contracted in the mortuary and made an award in the porter's favour. This award the Court of Appeal set aside, on the ground that the infection was not an accident within the meaning of the Act; and further that it was highly improbable—and in any case uncertain—that the infection was really contracted in the mortuary. From the lay point of view the latter conclusion is abundantly justified; indeed, it is impossible to believe, on the evidence submitted, that the man contracted infection in the mortuary, which was empty at the time. It appears that the porter had actually been in the wards on the day he cleaned the mortuary, and in any case it is probable that the actual infection occurred two or three days before the date assigned to it by the applicant's advisers. For hospital authorities the decision has far-reaching consequences which will be noted with relief by all institutional boards, and especially by those of hospitals for infectious disease.

